# Proteomic profile of dormant *Trichophyton Rubrum *conidia

**DOI:** 10.1186/1471-2164-9-303

**Published:** 2008-06-25

**Authors:** Wenchuan Leng, Tao Liu, Rui Li, Jian Yang, Candong Wei, Wenliang Zhang, Qi Jin

**Affiliations:** 1 State Key Laboratory for Molecular Virology and Genetic Engineering, Institute of Pathogen Biology, Chinese Academy of Medical Sciences, Beijing 100730, PR China; 2 National Institute for Viral Disease Control and Prevention, Chinese Center for Disease Control and Prevention, Beijing 100176, PR China; 3 Department of Basic Veterinary Science, College of Animal Science and Veterinary Medicine, Jilin University, Changchun, 130062, PR China

## Abstract

**Background:**

*Trichophyton rubrum *is the most common dermatophyte causing fungal skin infections in humans. Asexual sporulation is an important means of propagation for *T. rubrum*, and conidia produced by this way are thought to be the primary cause of human infections. Despite their importance in pathogenesis, the conidia of *T. rubrum *remain understudied. We intend to intensively investigate the proteome of dormant *T. rubrum *conidia to characterize its molecular and cellular features and to enhance the development of novel therapeutic strategies.

**Results:**

The proteome of *T. rubrum *conidia was analyzed by combining shotgun proteomics with sample prefractionation and multiple enzyme digestion. In total, 1026 proteins were identified. All identified proteins were compared to those in the NCBI non-redundant protein database, the eukaryotic orthologous groups database, and the gene ontology database to obtain functional annotation information. Functional classification revealed that the identified proteins covered nearly all major biological processes. Some proteins were spore specific and related to the survival and dispersal of *T. rubrum *conidia, and many proteins were important to conidial germination and response to environmental conditions.

**Conclusion:**

Our results suggest that the proteome of *T. rubrum *conidia is considerably complex, and that the maintenance of conidial dormancy is an intricate and elaborate process. This data set provides the first global framework for the dormant *T. rubrum *conidia proteome and is a stepping stone on the way to further study of the molecular mechanisms of *T. rubrum *conidial germination and the maintenance of conidial dormancy.

## Background

Infections caused by dermatophytes are widespread, are increasing in prevalence on a global scale, and have been considered a major public health concern in some areas of the world. Among dermatophytes, the dimorphic fungus *Trichophyton rubrum *represents the most clinically important species, which accounts for as many as 69.5% of all dermatophytoses in humans [1,2]. In addition to the well-known superficial infections caused by this organism, such as tinea capitis, tinea corporis, tinea inguinalis, tinea manus, tinea unguium, and tinea pedis, *T. rubrum *is also responsible for deep dermal invasion in immunocompromised patients [3-6]. Moreover, *T. rubrum *infections are often intractable, and relapses frequently occur after cessation of antifungal therapy [7]. The prevalence of *T. rubrum *infections and the anthrophilic nature of this species make it a good model for the study of pathogenic superficial fungi.

Conidia are a primary means of dispersion and provide a safe house for the filamentous fungal genome during adverse environmental conditions. Asexual sporulation is an important means of reproduction for *T. rubrum*. This species can produce numerous pear-shaped or club-shaped microconidia, which are thought to be the primary causative agents of skin and nail infections in humans [8,9]. Despite their importance in pathogenesis and physiology, the conidia of *T. rubrum *remain understudied. The studies on *T. rubrum *are mainly focused on epidemiology, clinical case reports, strain relatedness, and drug susceptibilities [9]. Little is known about its genetic and biological characteristics. Recently, gene expression during *T. rubrum *conidial germination was examined by microarray [10], providing insight into important biological and cellular events related to conidial germination. The study show that there is a pool of pre-existing mRNAs in dormant conidia that are important in maintaining dormancy and initiating germination [10]. However, we still know nothing about the conidial proteome during sporulation and conidial germination. For example, when searching PubMed using "*Trichophyton rubrum" *and "proteomics" as key words, the result returned is "No items found". The situation is extremely incommensurate to the prevalence of infections caused by this organism. Because of advances in proteomic technologies and the availability of a database of *T. rubrum *expressed sequence tags (ESTs) which contain 10224 unique ESTs [11,12], a global identification of proteins in its dormant conidia is achievable now.

In this study, we described the proteome of dormant *T. rubrum *conidia using a shotgun proteomics approach, an approach that was successful for large-scale proteomics analyses in several model organisms [13,14]. Combining the shotgun strategy with sample prefractionation and multiple enzyme digestion, we identified 1026 proteins from dormant *T. rubrum *conidia, which constitute the first global view of the proteome of dormant conidia of dermatophytes. These proteins covered nearly all major biological processes. Some spore specific proteins were identified. We hypothesize that these spore specific proteins are related to the survival and dispersal of *T. rubrum *conidia. We also identified many proteins that were important to conidial germination and the response to environmental conditions. The identification of proteins preserved in conidia may provide stepping stones on the way to further study of the molecular mechanisms of *T. rubrum *conidial germination and permit a better understanding of the maintenance of conidial dormancy. Since it has been proposed that the identification and targeting of conidial germination-specific processes provides an excellent strategy for drug and fungicide development in these pathogenic fungi [15], the present study may provide some clues for further approaches to finding new drug targets.

## Results and Discussion

### Assessment on the purity of prepared *T. rubrum *conidia and counting of microconidia and macroconidia

According to the numbers of conidia and hyphal fragments obtained from 10 independent counts of 1000-fold diluted conidial suspensions, the purity of prepared conidia was calculated as 99.8% (see additional file 1). This means that amount of conidia in our samples is three orders of magnitude higher than the contaminating hyphal fragments. When counting conidia microscopically, we found that microconidia were the predominant type of conidia produed by *T. rubrum *isolate BMU 01672 under conditions used in present study and accounted for approximate 99.6% of the conidia population. Most of microconidia were pear-shaped, while club-shaped microconidia were occasionally observed. Cylindrical- or cigar-shaped macroconidia were rarely seen in 10 independent samples. Detailed numbers of microconidia and macroconidia were shown in additional file 1.

### *T. rubrum *conidia protein identification

**Figure 1:**
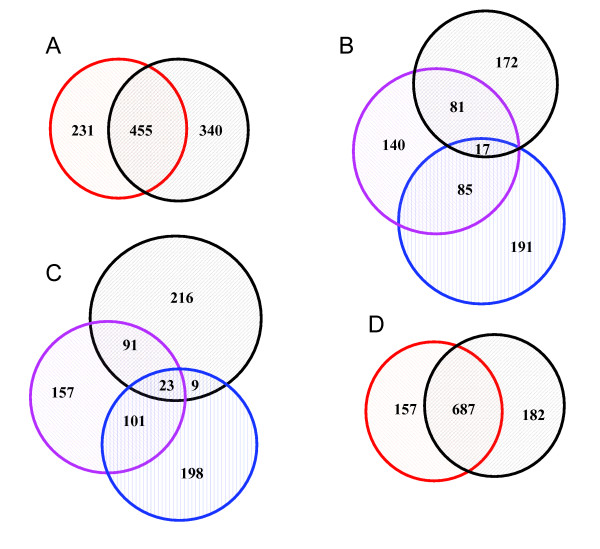
**The numbers of identified protein from different enzymatic digestion and sample prefractionation**. (A) Red circle represents the proteins identified by tryptic digestion and black circle indicates the proteins identified by proteinase K digestion. (B) The contribution of sample prefractionation to the identification of proteins digested by trypsin. Black circle represents the identified proteins from fraction of molecular weight less than 30 kDa. Pink circle represents the identified proteins from fraction of molecular weight between 30 and 50 kDa. Blue circle represents the identified proteins from fraction of molecular weight more than 50 kDa. (C) The contribution of sample prefractionation to identification of proteins digested by proteinase K. Black circle represents the identified proteins from fraction of molecular weight less than 30 kDa. Pink circle represents the identified proteins from fraction of molecular weight between 30 and 50 kDa. Blue circle represents the identified proteins from fraction of molecular weight more than 50 kDa. (D) The overlapping of identified proteins between duplicate analyses. Red circle represents the proteins identified by analysis 1 and black circle indicates the proteins identified by analysis 2.

A global view of the proteome of *T. rubrum *conidia was gained through a shotgun proteomics strategy combined with sample prefractionation and multiple enzymes digestion. After combining the MS/MS data generated from all twelve analyses, we were able to assign 5304 peptides to MS spectra leading to the identification of 1026 unique proteins from *T. rubrum *conidia. A complete list of the proteins and peptides identified is available as additional file 2. The overlap of identified proteins between duplicate analyses was approximately 67% (see Figure 1D). At present, the results from replicate analyses usually overlap by ~70% in most of proteomics studies, and protein and proteome coverage could be dramatically improved when samples were analyzed multiple times [16].

A study conducted recently suggested that multiple enzymatic digestions facilitated the detection of overlapping but distinct sets of proteins and provided a significant increase in the number of protein identifications in a complex proteomic sample [17]. Figure 1A shows the contribution of the two protease digestion approach, which was used in present experiments for protein identification. 686 and 795 proteins were respectively identified by using trypsin and proteinase K (PK) digestion. The numbers of proteins specifically identified by two different digestions was 571, accounting for 56% of the 1026 identified proteins. This showed that different enzymatic digestion can significantly improve proteome coverage, resulting in a more detailed map of the conidia proteome in this case.

The prefractionation can effectively reduce sample complexity thereby increasing detection sensitivity, which is necessary for identifying low abundance proteins [18]. Here, we adopted centrifugal ultrafiltration to divide the conidia proteome into three fractions based on molecular weight (*Mr*) of proteins. Of the 686 proteins identified by tryptic digestion, each fraction specifically contributed 191, 140, and 172 proteins (Figure 1B). This collection of proteins accounted for 73% (503/686) of total proteins identified by tryptic digestion. Similarly, proteins specifically identified from the three fractions accounted for 71% (571/795) of all proteins identified by PK digestion (Figure 1C). These results suggest that prefractionation by centrifugal ultrafiltration can significantly increase the number of identified proteins. Although we could not obtain statistics on the *Mr *distribution of identified proteins in different fractions due to a lack of full length cDNA sequences, Figure 1B and 1C clearly showed that the samples were effectively separated by centrifugal ultrafiltration.

Although trace contamination in conidial samples may lead to the possibility of a very few proteins resulting from hyphal fragments, it is impossible to determine which identified proteins belong to hyphal fragments at present due to lack of protein quantitative information in this work and hyphae-specific proteins database. Furthermore, the purity of prepared conidia is high enough to guarantee the reliability of the analysis results.

### Comparison to non-redundant, eukaryotic orthologous groups, and gene ontology databases

To obtain as much functional annotation information as possible, the identified proteins were compared to those in the NCBI non-redundant (NR) protein database, the eukaryotic orthologous groups (KOG) database, and the gene ontology (GO) database (see additional file 3). The comparison with the NR database enabled us to assign putative functions to or find homologues from other organisms for 857 (83%) identified proteins. The remaining 169 (17%) proteins were only weakly similar or not similar to known sequences (E ≥ 1E-05).

**Figure 2:**
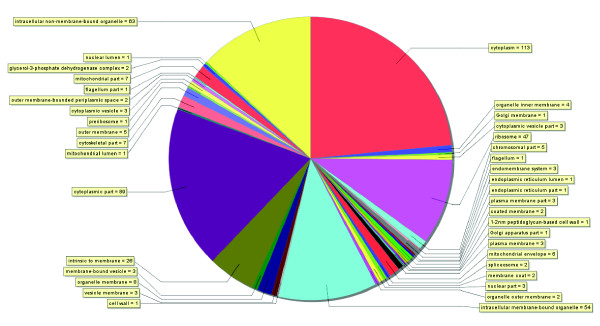
**Gene Ontology (GO) cellular component annotation for identified proteins**. The GO cellular component categories are shown as a pie-chart, and the numbers in parentheses represent the proteins included in each term. The figure was produced using Blast2GO.

Comparison with the GO database provided more information on the cellular components and the biological processes of the identified proteins. Annotation information was obtained for 630 (61%) of the identified proteins through GO database comparison, and the cellular distribution of these proteins was shown in Figure 2. It was clear that the identified proteins were predicted to cover all main organelles such as nucleus, mitochondrion, plasma membrane, Golgi apparatus, and endoplasmic reticulum, indicating a reflective sampling of the *T. rubrum *conidia proteome.

Proteins were sorted into main functional classes based on the KOG database comparison. 645 (63%) of the identified proteins fell into various functional categories, while 381 (37%) of the proteins had no related KOG based function. Functional classification of the identified proteins is summarized in table 1, where almost all categories and major biological processes are represented. This indicates a complex profile of the *T. rubrum *conidial proteome. Furthermore, the complexity of the conidial protein profile suggests that these dormant bodies are not merely a warehouse preserving the genome, and that the maintenance of conidial dormancy may be a considerably intricate and elaborate process. Besides maintaining the quiescent status of conidia, these proteins may also be involved in conidiation and conidial germination.

**Table 1:** Functional classification of identified proteins in *T. rubrum *conidia based on the KOG database

Functional Classification	Numbers of ID	Percents in Total
Information storage and processing		

Translation, ribosomal structure and biogenesis	101	9.8%
RNA processing and modification	30	2.9%
Transcription	20	1.9%
DNA replication, recombination and repair	7	0.68%
Chromatin structure and dynamics	30	2.9%

Cellular Processes and Signalling		

Cell cycle control, cell division, chromosome partitioning	7	0.68%
Defence mechanisms	1	0.1%
Signal transduction mechanisms	22	2.1%
Cell wall/membrane/envelope biogenesis	7	0.68%
Cytoskeleton	16	1.6%
Extracellular structures	4	0.38%
Intracellular trafficking, secretion, and vesicular transport	20	1.9%
Posttranslational modification, protein turnover, chaperones	71	6.9%

Metabolism		

Energy production and conversion	71	6.9%
Carbohydrate transport and metabolism	43	4.2%
Amino acid transport and metabolism	45	4.4%
Nucleotide transport and Metabolism	12	1.2%
Coenzyme transport and Metabolism	12	1.2%
Lipid transport and metabolism	21	2.1%
Inorganic ion transport and Metabolism	21	2.1%
Secondary metabolites biosynthesis, transport and catabolism	15	1.5%

Poorly characterized		

General function prediction only	69	6.7%
Function unKnown	19	1.9%

Anonymous NO related KOG	381	37.1%

Total	1026	

ID indicates all proteins identified in our experiments.

### Cell wall proteins and wall biosynthesis related proteins in conidia

The fungal cell wall is a complex structure composed typically of chitin, 1,3-β- and 1,6-β-glucan, and mannoproteins. Both conidial and mycelial cell walls contain 1,3-β-glucan, chitin, and mannoproteins, but the percentage of respective components present in the cell walls of the two types of cells can be different [19]. In dormant *T. rubrum *conidia, according to NR or KOG annotations, we identified 22 proteins that were components of the cell wall or were related to cell wall biosynthesis or remodeling (see additional file 4). The presence of the 1,3-β-glucan synthase catalytic subunit, chitin synthase, and mannosyltransferases in conidia suggests that cell wall synthesis or reorganization may be considerably active during sporulation in *T. rubrum*, and that the cell wall of *T. rubrum *conidia, similar to other filamentous fungi, is composed of 1,3-β-glucan, chitin, and mannoproteins.

Protein S11555 exhibits high homology to the protein sequence of Ecm33 (E-value, 2.04E-71) which is a glycosylphosphatidylinositol-anchored cell wall organization protein. Ecm33 is found in all fungal genomes sequenced to date, and its orthologs are essential for sporulation in yeast [20-22]. A recent study showed that Ecm33 influenced conidial cell wall biosynthesis in *Aspergillus fumigatus *[22]. Disruption of Ecm33 resulted in several morphogenetic aberrations, including: (1) a defect in conidia separation, (2) an increase in diameter of conidia associated with an increase in cell wall chitin concentration and (3) conidia that were sensitive to the absence of aeration during long-term storage. These results suggest that in *T. rubrum*, Ecm33 may also play some roles in conidiation, conidial cell wall biosynthesis, and viability of conidia in adverse environmental conditions.

The surface of many fungal conidia is covered by a thin layer of regularly arranged rodlets which are mainly proteinaceous [23,24], and favor air buoyancy and dispersion of the conidia by air currents [25]. The proteins responsible for this rodlet configuration are hydrophobins. The rodlet layer of the conidia of *Neurospora crassa*, *Beauveria bassiana*, and *Magnaporthe grisea *only contain a single type of hydrophobin [26-28]. *Aspergillus nidulans *and *A. fumigatus *have two conidial hydrophobins, which are *rodA *and *dewA*, *rodA *and *rodB*, respectively [29-31]. In *T. rubrum *conidia, we identified a protein (S11382) that is homologous to conidial hydrophobin RodB (E-value, 5.82E-07), but did not find any protein corresponding to RodA. Based on these results, we hypothesize that the surface of *T. rubrum *conidia should have a hydropbobic rodlet layer which facilitates its dispersal by air.

### Protein synthesis in *T. rubrum *conidia

Previous biochemical and genetic evidence suggests that protein synthesis is essential for spore germination in fungi, and that conidial germination cannot take place when protein synthesis is blocked [19,32-36]. Translation was one of the first measurable effects (<20 min) during conidial germination [37]. In dormant *N. crassa *conidia, high levels of free ribosomes were observed, which in the presence of a carbon source, associated with a pre-existing pool of mRNA within 15 min to form polysomes [38].

In our result, 101 proteins were assigned to "translation" based on KOG classification, and accounted for 10% of total identified proteins in *T. rubrum *conidia (see additional file 5). This suggests that proteins involved in translation are relatively abundant in dormant *T. rubrum *conidia. Of 101 proteins, 56 (more than 50%) were structural components of cytosolic ribosome. As mentioned above, a pool of mRNAs are preserved in dormant *T. rubrum *conidia. When encountering appropriate conditions, such as the presence of a carbon source, these ribosomes could rapidly associate with preserved mRNAs to produce proteins required for conidial germination. Aside from the structural components of cytosolic ribosomes, we also identified translational initiation factors, elongation factors, and aminoyl-tRNA synthases in the *T. rubrum *conidia proteome. In *A. nidulans *[32], *sgdA*, *sgdB*, and *sgdC *respectively encode translation initiation factor eIF3 subunit, seryl-, and cysteinyl-tRNA synthase and the phenotypes of these *sgd *mutants were characterized. At the restrictive temperature, there was no conidial germination in any of the *sgd *mutants.

In addition to proteins related to cytoplasmic protein synthesis, we also identified some proteins involved in mitochondrial protein synthesis in *T. rubrum *conidia, including nine structural components of the mitochondrial ribosome, one component of mitochondrial translation elongation factor, and two components of mitochondrial polypeptide chain release factor. Unlike the system of cytoplasmic protein synthesis, mitochondrial protein synthesis appears not to be essential for spore germination in some fungi. In *N. crassa *and *Botryodiplodia*, chloramphenicol and ethidium bromide (inhibitors of mitochondrial protein synthesis) did not block conidial germination [39,40]. In *T. rubrum*, the necessity of mitochondrial protein synthesis for conidial germination has not been evaluated and needs to be further investigated.

### Dormant *T. rubrum *conidia metabolism profile

Although dormant conidia are in quiescent status which means low metabolic activities and rates of oxygen consumption [19], we identified many proteins belonging to the glycolysis system, subunits of the pyruvate dehydrogenase complex, the tricarboxylic acid cycle, and the oxidative phosphorylation machinery in *T. rubrum *conidia (see additional file 6). These results indicate that proteins involved in energy production and carbohydrate metabolism are retained in dormant *T. rubrum *conidia. Early studies demonstrated that spore germination was dependent on the function of the standard, cytochrome-mediated electron transport system in *Botryodiplodia theobromae *and *Neurospora crassa *[39,40]. The presence of cyanide (an inhibitor of this pathway) resulted in a complete block of spore germination. In dormant *T. rubrum *conidia, some components of all five respiratory chain complexes were identified, including six subunits of complex I (NADH-CoQ reductase), one subunit of complex II (Succinate-CoQ reductase), five subunits of complex III (CoQ-Cytochrome C reductase), three subunits of complex IV (Cytochrome C oxidase), and 11 subunits of complex V (F1F0-type ATP synthase). This was consistent with dormant *N. crassa *conidia where all of the enzyme components in this standard pathway, such as cytochrome c oxidase and F1F0-type ATP synthase, appeared to be assembled and preserved [39]. These results indicate that the cytochrome-mediated electron transport system in *T. rubrum *conidia may be important for conidia germination, just as in *Botryodiplodia *and *N. crassa*.

In fungal spores, trehalose may account for up to 15% of the dry mass and has been proposed to serve as a stress protectant [37]. Systematic mobilization of the trehalose pool is one of the first measurable effects during spore germination, which suggests that it may also act as a reserve carbohydrate needed at the onset of this particular developmental stage [41]. Two *T. rubrum *conidia proteins, S00116 and S04712, were involved in trehalose biosynthesis. They were annotated as trehalose-6-phosphate synthase component TPS1 and trehalose synthase *ccg-9 *respectively. In *S. cerevisiae*, glucose-6-phosphate plus UDP-D-glucose is converted to α,α-trehalose-6-phosphate by TPS1. Then the latter is converted to trehalose by TPS2 [42]. *ccg-9 *is trehalose synthase of *N. crassa *and its mRNA peaks at the time of initiation of conidiation [43]. A *ccg-9 *null mutant produced abnormal morphological spores, suggesting that *ccg-9 *is involved in developmental morphogenesis of the asexual conidiospores. This evidence implies that trehalose is also important for *T. rubrum *conidia and may be implicated in conidial morphogenesis, as observed in *N. crassa*.

### Signalling transduction proteins in dormant conidia

In *T. rubrum *conidia, we identified 32 proteins that were related to a variety of signalling transduction systems such as components of Ras-related GTPase pathway, the cAMP-PKA pathway, the calcium signalling pathway, the G-protein pathway, the two-component signal transduction system, and some serine/threonine protein kinase/phosphatases and signal histidine kinases (see additional file 7). This reflects the potential complexity of signalling transduction networks in *T. rubrum *conidia.

Currently, three signalling transduction pathways (calcium signalling, Ras/MAPK pathway and cAMP-PKA pathway) have prompted intensive efforts on their roles at early stage of spore germination [33]. In various fungi, roles of these signalling events in spore germination seemed to be conflicting and variable across species [33,37]. Despite the conflicting results from previous studies, once these signalling pathways participated in spore germination, they were usually involved in mediating carbon source sensing, the transition from isotropic to polarized growth, activating metabolic activities, transcription, or protein synthesis [32,37,44]. The study of the transcriptional patterns on conidia germination in *T. rubrum *[10] showed that the Ras-related GTPase and cAMP-PKA pathways may play roles in conidial germination, and that the two-component signal transduction system may participate in response to changes in extracellular osmolarity during conidial germination. The precise roles of these signalling transduction pathways in maintaining the dormant state of conidia in *T. rubrum *needs to be studied further.

### Environmental stress resistance proteins in dormant conidia

Resistance to adverse environmental conditions, such as desiccation, heat, and osmotic stress, is one of the most impressive properties of dormant conidia. For example, dry conidia can be stored for a long time without loss of viability, and more than 90% of dehydrated conidia can survive after being heated to 124°C for 3 min[19]. These properties permit conidial survival and dispersal in adverse environments. Consistent with these characteristics, we identified 58 proteins related to response to various environmental stresses in *T. rubrum *conidia according to GO annotation, including desiccation-, heat-, cold-, oxidative stress-, osmotic stress-, starvation-, drug-, salt stress-, unfolded protein-, metal ion-, DNA damage- and general stress-related proteins (see additional file 8). The classification of these stress related proteins was somewhat overlapping.

Of 58 proteins, S03304, S12066, and S13929 were implicated in sporulation or conidium formation according to GO annotation. S03304 and S12066 were annotated as vacuolar protease B and ubiquitin-like protein by GO, corresponding to PRB1 and UBI4 of *S. cerevisiae *(E-value, 1.2E-115 and 5.0E-108) respectively. PRB1 is one of the major proteolytic catalysts in yeast cell under cellular response to starvation, and cells deficient in PRB1 showed a considerably reduced sporulation frequency [45]. UBI4 is required by yeast cells for resistance to stress conditions [46]. Although the ubi4/UBI4 diploids formed viable spores initially, these spores lost viability rapidly. And the ubi4/ubi4 diploids were sporulation defective [46]. S13929 was the homologue of catalase1 of *N. crassa *(E-value, 2.9E-75). *N. crassa *possessed three catalases (catalase1 to catalase3) which served as hydrogen peroxide scavenging enzymes and were differentially expressed during the asexual life cycle [47,48]. Catalase3 activity increased at the end of exponential growth, catalase2 activity rose transiently in the aerial hyphae, and catalase1 activity augmented many times during formation of conidia. Catalase1 was found in conidia at a very high concentration and exhibited an unusual resistance to inactivation by temperature and various denaturants. These characteristics make catalase1 especially suitable for the spore and its survival.

### Comparison of dormant conidia proteome with transcriptome

In our previous study on gene expression during *T. rubrum *conidial germination [10], we identified pre-stored mRNAs of 560 genes in dormant conidia. These genes showed maximum expression levels in dormant conidia and then expression levels of most of them dropped quickly at the onset of germination. Among 560 genes, annotation information was obtained for 336 through GO database comparison. Functional annotation suggested that these pre-stored mRNAs covered wide range of biological processes, including "response to external or abiotic stimulus", "transcription", "regulation of biological process", "cell communication", "development", and "metabolism", "protein synthesis", and "signalling transduction" etc. These transcriptional analysis results were consistent with our proteomic results that revealed the complexity of dormant conidia proteome.

Of 560 genes, proteins corresponding to 59 genes were identified by the present proteomic analysis (see additional file 9). mRNA levels of 43 genes (73%) were more than 2-fold of reference mRNA levels, suggesting their relative high expression levels in dormant conidia [10]. Other proteins corresponding to remaining genes were not detected in present study. This indicates that either they may be extremely low abundant proteins that can not be detected or they are only synthesized at the activation of germination. We lack direct evidence to assert whether the 59 identified proteins are the translational products of pre-stored mRNAs in dormant conidia or have existed during sporulation. However, in view of high expression levels of these pre-stored mRNAs in dormant conidia and the existence of active polysomes in ungerminated conidia [38], at least partially, these proteins may be the translational products of pre-stored mRNAs in dormant conidia. According to functional annotation obtained from NR, GO, and KOG database comparison, the 59 proteins were involved in "response to external stress or stimulus", "RNA processing and modification", "transcription regulation", "chromatin structure and dynamics", "metabolism", "signalling transduction", "protein synthesis", "posttranslational modification", and "cell wall biosynthesis". These proteomic analysis results support the conclusions drawn from our previous study that maintenance of dormancy and activation of germination are complicated, and need the participation of various pathways. The attachment of dormant conidia to human skin is essential for establishment of dermatophytes infection. Of the 59 proteins, S12031 and S03654 may be involved in pathogenicity of dormant conidia. S12031 is a homologue of yeast HSP12 which is essential for biofilm formation and related to cell adhesion [49]. S03654 is a homologue of the Aspergillus clavatus NRRL 1 adhesin according to NR database comparison. The two proteins may be related to attachment of conidia to human skin cell, and are potential virulence factors of *T. rubrum *conidia.

## Conclusion

We provided the first global view of the *T. rubrum *conidia proteome by combining shotgun proteomics with sample prefractionation and multiple enzymes digestion. Our results show that the proteome of *T. rubrum *conidia is of considerable complexity, and that proteins involved in nearly all biological processes are preserved in conidia. These results suggest that the maintenance of conidial dormancy is an intricate and elaborate process. Conidia are in a dormant, but competent, state. And germination could be readily initiated when conidia encounter the appropriate environmental conditions. This study provides an important dataset that can be used for further understanding the maintenance of dormancy and conidial germination that are important but understudied stages in fungi life cycles.

## Methods

### Reagents

All reagents were purchased from Sigma-Aldrich Co. (St. Louis, Missouri) with the exception of HPLC grade acetonitrile (ACN) and formic acid, which were purchased from Merck KGaA (Darmstadt, Germany). Modified sequencing grade trypsin and proteinase K (PK) were purchased from Roche Diagnostics Co. (Indianapolis, Indiana).

### Strains and culture conditions

The *T. rubrum *clinical isolate BMU 01672 used in present experiments was obtained from Professor Ruoyu Li (Research Center for Medical Mycology, Peking University). The isolate was confirmed as *T. rubrum *by morphologic identification, as well as by PCR amplification and sequencing of the 18S ribosomal DNA and internal transcribed spacer (ITS) regions.

*T. rubrum *was cultured for 2–3 weeks at 28°C on potato glucose agar (Difco) to produce conidia. A mixture of conidia and hyphal fragments was harvested from the medium at 4°C in distilled water and then filtered through a 70 μm pore size nylon filter twice to remove hyphal fragments. To assess the purity of prepared conidia, filtered conidia suspension was serial diluted 1000 fold with distilled water, and the diluted conidia suspension was independently counted 10 times on a hemacytometer under the Nikon microscope (Nikon Corp., Tokyo, Japan) to determine the numbers of microconidia, macroconidia and hyphal fragments. Obtained numbers of conidia and hyphal fragments from 10 independent counting were used for calculating the purity of filtered conidia. The filtered conidia suspension were collected by centrifugation at 1800 g for 10 min and washed twice with ice-cold distilled water. The collected conidia were frozen at -80°C for subsequent experiments.

### Cell lysis and fractionation

Conidia were ground thoroughly in liquid nitrogen with a mortar and pestle into powder, which was transferred to liquid nitrogen-cooled 15 ml falcon tubes. Two volumes of lysis buffer (phosphate buffered saline pH 7.4, 8 M urea) were added to the powder and the mixture was vortexed vigorously for 10 min at 4°C. The lysate was held on ice for 30 min and centrifuged at 10000 g for 20 min at 4°C. The clarified supernatant was then transferred to a new 15 ml tube. Protein concentration in the supernatant was measured by BCA assay (Pierce, Rockford, IL). Due to the wide dynamic range of individual proteins in cells, which presumably varies over five to six orders of magnitude, a key requirement for a comprehensive proteome analysis is to reduce sample complexity so that access to low abundance proteins can be achieved. For this purpose, the supernatant was separated into three fractions by centrifugal ultrafiltration with Microcon filters (Millipore, Billerica, MA) according to molecular weight (*Mr*) size, including less than 30 kDa, between 30 and 50 kDa, and more than 50 kDa. The protein concentration of each fraction was measured by BCA assay.

### Sample digestion

For PK digestion, each fraction (500 μg protein) was solubilized at pH 11.5 in Na_2_CO_3 _and 8 M urea, and then samples were digested following a protocol for MudPIT analysis [50]. For trypsin digestion, each fraction (500 μg protein) was adjusted the pH to 8.5 with 1 M NH_4_HCO_3 _and solubilized in 8 M urea, and cleaved following Washburn's protocol [13]. After digestion, samples were filtered through 10 kDa cutoff filters (Microcon YM-10, Millipore) and desalted with solid phase extraction column (HLB 3 cc, Waters) following the manufacturer's protocol. All peptide fractions were concentrated by a Speed-vac centrifuge (Savant) and dissolved in 0.1% formic acid for the subsequent 2D LC-MS/MS analysis.

### Shotgun proteomics

Orthogonal 2D LC-MS/MS analysis was performed using a ProteomeX Workstation (Thermo Finnigan), which consists of a strong cation exchange column (BioBasic SCX, Thermo Hypersil-Keystone) and two parallel C_18 _reversed-phase microcapillary columns (BioBasic-18, 100 × 0.18 mm, Thermo Hypersil-Keystone). The solutions used were as follows: 5%ACN/0.1% formic acid (for mobile phases A&C), ACN/0.1% formic acid (for mobile phase B). A sample peptide digest was loaded onto the SCX column and 200 uL applications of NH_4_Cl in increasing concentrations (5, 10, 15, 20, 25, 30, 35, 40, 45, 50, 60, 70, 80, 100, and 500 mM) were used to elute peptide fractions from the SCX column onto the RP column, respectively. All reversed-phase separations were synchronized with a 140 min gradient. The RP gradient program was along three successively linear gradients: a 30-min gradient to 20% mobile phase B followed by a 60-min gradient to 40% mobile phase B and then a 20-min gradient to 90% mobile phase B. The flow rate through the column was 2 μL/min.

Eluting peptides were electrosprayed into the LCQ mass spectrometer with a distally applied spray voltage of 3.4 KV, and the capillary transfer temperature was set at 180°C. The RP column eluate was continuously analyzed during the entire 15-step chromatography program. In data-dependent acquisition, the three most intense precursor ions detected in the full range mass survey (300–1500 m/z) were selected and fragmented with dynamic exclusion for 60 s. MS/MS was triggered by a minimum signal threshold of 10000 counts at 35% normalized collision energy. Both HPLC pump and the mass spectrometer were controlled by the Xcalibur software (Thermo Electron). All samples were analyed in duplicate.

### Data evaluation

From raw files, MS/MS spectra were exported as individual files in DTA format using extract_msn.exe program contained within Bioworks v3.2 software (Thermo Electron) under the following settings: peptide mass range: 500–4000; minimal total ion intensity threshold: 10000; minimal number of fragment ions: 15; precursor mass tolerance: ± 1.4 amu; group scan: one; with a minimum group count of one.

Our group has established a database of ESTs for *T. rubrum *which contains 10224 unique ESTs [11,12]. 69340 proteins sequences with minimal length 50 amino acids were obtained from the translated six reading frames of 10224 ESTs. The protein searching database used in present study was based on these protein sequences and was composed of two parts. The target component was composed of these protein sequences plus protein sequences of trypsin, PK, and keratin, and the decoy component was composed of the reversed sequences of all proteins in the target protein sequence database. All MS/MS data were batch searched against this target/decoy database using SEQUEST algorithm contained within Bioworks v3.2 software [51]. The following parameters were used in searching process: peptide mass tolerance was set as 2.0 amu; fragment ion tolerance was 0.5 amu; cysteine residues were searched as carbamidomethylated (mass increment of 57.02146 Da); for tryptic digestion, one missed cleavages was allowed; for digestion by PK, no enzyme was selected. An accepted SEQUEST was used with a ΔCn (delta normalized correlation) cutoff of at least 0.1 regardless of charge state. The minimum cross correlation coefficients (Xcorr) of 1.7 and 1.9 for a + 1 proteolytic peptide by trypsin and PK, 2.2 and 2.5 for a + 2 proteolytic peptide by trypsin and PK, and 3.3 and 3.75 for a + 3 proteolytic peptide by trypsin and PK, respectively, were proposed to meet a peptide identification false discovery rate (FDR) of ~1% threshold. The FDR was estamited using the target/decoy database approach [52]. Identification of individual proteins matched with two or more unique peptides was just considered as valid identification.

### Bioinformatics analysis

In order to obtain information of functional annotation of identified proteins, the nucleotide sequences of all identified proteins were compared to the NCBI non-redundant (NR) protein database (01/2007) and the gene ontology (GO) database (01/2007) [53] using Blast2GO software (Version 1.6) [54] and to eukaryotic orthologous groups (KOG) database (03/2007) [55] by BlASTX. The threshold for cutoff were E<1 E-05.

## Abbreviations

ΔCn: delta cross-correlation; EST: expressed sequence tag; FDR: false discovery rate; GO: gene ontology; KOG: eukaryotic orthologous groups database; MudPIT: Multidimensional Protein Identification Technology; NR: the NCBI non-redundant protein database; PK: proteinase K; SCX: strong cation exchange column; Xcorr: cross-correlation coefficients.

## Authors' contributions

WL carried out collection of *T. rubrum *conidia, samples preparation and prefractionation, samples analysis by mass spectrometer, data processing and bioinformatics analysis, and drafted the manuscript. TL contributed to samples analysis by mass spectrometer, data processing and bioinformatics analysis. RL performed samples preparation, protein prefractionation, and samples analysis by mass spectrometer. JY contributed to construction of protein searching database. CW and WZ participated in sample analysis by mass spectrometer. QJ proposed the research goal, supervised the whole studies and provided a critical review of the manuscript. All authors read and approved the final manuscript.

## Supplementary Material

Additional file 1 Results of conidia counting and assessment of the purity of conidia sample. Click here for file

Additional file 2 A complete list of the identified proteins and peptides of *T. rubrum *conidia in present study. Click here for file

Additional file 3 Functional annotation information for the identified proteins of *T. rubrum *conidia. Click here for file

Additional file 4 Cell wall proteins and wall biosynthesis related proteins in conidia. The NR, KOG, and GO annotation information for these proteins was included in this supplementary table. Click here for file

Additional file 5 The identified proteins of conidia involved in translation. The NR, KOG, and GO annotation information for these proteins were included in this supplementary table. Click here for file

Additional file 6 Identified proteins of conidia involved in glycolysis, the tricarboxylic acid cycle, and the oxidative phosphorylation. The NR, KOG, and GO annotation information for these proteins were included in this supplementary table. Click here for file

Additional file 7 Signalling transduction proteins in dormant conidia. The NR, KOG, and GO annotation information for these proteins were included in this supplementary table. Click here for file

Additional file 8 Environmental stress resistance proteins in conidia. The NR, KOG, and GO annotation information for these proteins were included in this supplementary table. Click here for file

Additional file 9 The identified proteins corresponding to 59 pre-stored mRNAs in dormant conidia. The NR, KOG, and GO annotation information for these proteins were included in this supplementary table. Click here for file
